# From care practices to speculative vignettes–design considerations for robots in good care

**DOI:** 10.3389/frobt.2024.1347367

**Published:** 2024-08-09

**Authors:** Ralf Vetter, Anna Dobrosovestnova, Helena Anna Frijns, Laura Vogel, Katharina Brunnmayr, Christopher Frauenberger

**Affiliations:** ^1^ Human-Computer Interaction Division, Department for Artificial Intelligence and Human Interfaces, University of Salzburg, Salzburg, Austria; ^2^ Institute of Visual Computing and Human-Centered Technology, TU Wien, Vienna, Austria; ^3^ Institute of Management Science, TU Wien, Vienna, Austria

**Keywords:** good care, robots, field studies, care-centered value sensitive design, reflexive thematic analysis, vignettes, speculative design, design considerations

## Abstract

The care sector has become one of the test beds for developing robotic technologies, which have been promised to mitigate problems with aging populations and labor shortages. Despite these promises, the practical application of such technologies have been met with limited success. Apart from technical limitations, other challenges exist in the way we approach designing these technologies. Critical to the development in the care sector is understanding the complexity of the contexts, the needs and goals of diverse actors, and how these are socio-materially scaffolded. This paper presents a study conducted at the intersection of a value sensitive design and speculative design to understand these sensitivities. Based on the data collected in interviews (n = 6) and card workshops (n = 6) from care workers and residents in mobile care and care home contexts in Austria, we developed five themes capturing situated practices and understandings of good care as built on trust-developing routines, negotiations between different actors, affective and reciprocal dimension of care, care worker self-care, and material mediations. Subsequently, we created six speculative vignettes which serve as rhetorical devices to emphasize the tensions that arise with any technological intervention entering and reshaping existing care practices and relations. We argue that our approach can support robot designers to develop a rich understanding of the values and tensions in the specific context under study from the before design and development begin.

## 1 Introduction

Older adult care in countries of the Global North faces two interrelated challenges. First, the number and proportion of older adults in societies worldwide are increasing due to increasing life expectancy and improved medical treatment. This development is especially prevalent in countries in Europe and Northern America, where the percentage of people over 65 years of age is projected to increase from 18.7% in 2022 to 26.9% in 2050 (United Nations Department of Economic and Social AffairsPopulation Division, 2022). Aging is accompanied by various individual changes in intrinsic, i.e., physical and mental, capacities. To prevent a decline in functional abilities (e.g., mobility, meeting basic needs), older adults require supportive environments that help maintain intrinsic capacities or compensate for their loss (World Health Organization, 2020). One measure to support older adults with changes and challenges is providing professional care by care institutions. However, as a second challenge, while the number of older adults is increasing, the number of care workers (CWs) is decreasing due to the forthcoming retirement of cohorts with high birthrates. Predictions indicate a substantial shortage of professional carers in the coming decade (European Commission, 2022).

Together, the developments discussed above threaten the provision of good care. In response, policymakers and NGOs have identified digital technologies as one lever to provide qualitative care in the future. Among many technological interventions in development to this call are robots (World Health Organization, 2020; European Commission, 2022). Robots are envisioned to provide a variety of functionalities: lifting and mobilizing care recipients (CRs) (Mukai et al., 2010)), engaging in physical exercise (Martín Rico et al., 2013; Martinez-Martin and Cazorla, 2019), as affective companions (Hung et al., 2019), or to support people with dementia (Ghafurian et al., 2021).

Despite the potential as a helpful tool in care, the adoption rates of existing products and platforms remain lower than expected (Ienca et al., 2016; Östlund et al., 2023). Among the reasons for low acceptance are that existing technologies are far from resembling agile and versatile robots depicted in futuristic visions (Weiss and Spiel, 2022). Additionally, a lack of training (Melkas et al., 2020) and neglect of contextual circumstances in the design and development process (Östlund et al., 2023) contribute to low acceptance.

With this work, we address a neglect of contextual circumstances and follow calls in HRI for more direct stakeholder involvement and mutual learning between stakeholders and researchers to create meaningful robotic technologies for everyday life (e.g., Weiss and Spiel, 2022). Our study is embedded in a trans-disciplinary research project centered on a collaboration between research institutions and a care practice partner, a non-profit care provider in Austria. What we present here is a first step to an ongoing effort to develop novel robots for care. As such, this work aligns itself with a Participatory Design (PD) approach (Lee et al., 2017; Bødker et al., 2022) and takes inspiration from Value Sensitive Design (VSD) (Friedman and Hendry, 2019), in particular the care-centered value sensitive design (CCVSD) methodology (van Wynsberghe, 2013) to consider values - as the lived experiences of what is meaningful to care stakeholders - in the design of robotic technologies. Our studies were conducted in care homes and mobile care contexts operated by our partner institute.

Two research questions guide the approach of this study. Research Question 1 (RQ1) asks *“What does good care mean for care workers and care recipients in contexts of mobile care and care homes?”* To explore RQ1, as a phase of research-for-creation (Chapman and Sawchuk, 2012), we first conducted interviews and card workshops with CWs and CRs in mobile care and a care home. Based on the collected data, we developed five themes (*Building a trustful care relationship through time and routines*, *Negotiations of care between care stakeholders*, *Emotional care and reciprocity*, *Self-Care of Care Workers*, and *Socio-material Mediation of Care*) following a reflexive thematic analysis (RTA) (Braun and Clarke, 2022). The results are presented in Section 4.

Research Question 2 (RQ2) asks *“What should we consider in the design of robots based on identified meanings of good care?”* In response to RQ2, we performed a creation-as-research phase (Chapman and Sawchuk, 2012) and generated speculative vignettes of robotic technologies in care. We use the format of speculative vignettes as a method to illustrate how robots in care scenarios inevitably exert influence to tensions and themes of good care and articulated considerations by juxtaposing the vignettes with themes of good care in Section 5.

This article contributes to the ongoing efforts to design meaningful and desired care robots in two ways. First, we flesh out understandings of good care based on situated practices and values of care workers. Second, grounded in our speculative vignettes, we exemplify potential tensions but also spaces of possibilities for robot-mediated care.

The article is structured as follows: first, we present related work on socially assistive robots in healthcare, and value sensitive design and speculative design approaches. Then, the methodology of field studies, data analysis, and the process of writing speculative vignettes are presented. In Section 4, we present the themes developed in our reflexive thematic analysis. This is followed by speculative vignettes and related discussions 5. We conclude with limitations of our approach and opportunities for future work.

## 2 Related work

### 2.1 Socially assistive robots in health and care

Robot platforms such as NAO, Pepper, Moxi, ROBEAR and others have been applied for tasks ranging from therapy to delivery services, patient lifting, disinfection, rehabilitation, (Kyrarini et al., 2021), for therapeutic assistence and engagement of people with dementia (Ghafurian et al., 2021), and as companions for exercising, games, playing music, or walking (Yuan et al., 2023). Acceptability of these technologies remains an open challenge, for instance due to replacement fears Kyrarini et al. (2021). Further reasons for limited success are the highly individual nature of (dementia) care (Ghafurian et al., 2021), or accessibility issues, additional burdens to care workers, and reduced caregivers’ autonomy (Yuan et al., 2023).

To overcome outlined shortcomings, robot designers and developers are encouraged to become aware and directly implement the needs, expectations, resources and values of care workers and care recipients in design processes (Yuan et al., 2023). Participatory design approaches (Lee et al., 2017), and value sensitive design approaches (van Wynsberghe, 2013; Cheon and Su, 2016) considering the life-worlds of potential users in the design process are potential paths to achieve these sensitivities (Ghafurian et al., 2021).

### 2.2 Value sensitive design and care-centered value sensitive design

Value sensitive design is a methodology well established in Human-Computer Interaction aiming to consider people’s values in the design of technologies. Part of the VSD methodology is to empirically investigate the values of stakeholders (van Wynsberghe, 2013; Friedman and Hendry, 2019) developed the care-centered framework (CCF) and care-centered value sensitive design (CCVSD) methodology to explicitly address the design of robotic technologies in consideration of values. The CCVSD methodology builds on an investigation of the components context, care practice, actors, type of robot, and four values of care (attentiveness, responsibility, competence, and reciprocity, based on a care ethics theory by (Tronto, 1993)). The author suggests a prospective application aiming *“[…] to illuminate the relationship between the technical content of a care robot and the resulting expression of care values within a care practice.”* (van Wynsberghe, 2013, p. 410).

Despite being a widely acclaimed work, no empirical study following the CCVSD methodology exists to our knowledge. Related work either extended the CCVSD framework (e.g., by adding the dimension of touch (Grobbel et al., 2019)) or considered it in developing an alternative framework for healthcare technologies (e.g., (Poulsen and Burmeister, 2019; Jacobs, 2020)). Applied design processes of robotic technologies refer to it only as related work (e.g., (Casey et al., 2016)) but do not follow the proposed CCVSD methodology. A recent literature review (Yuan et al., 2023) draws on Tronto’s ethics of care framework (Tronto, 1993) and the CCF framework (van Wynsberghe, 2013) to analyze qualitative data from user studies with humanoid robots in residential care. Yuan et al. (2023) outline design principles on the basis of the reviewed projects. In contrast to approaches that involve adding an additional reflective layer on top of principles that are already abstract, we argue that it is important for robot designers to have a rich understanding of the specific context under study.

To address this research gap, our work directly applied the CCVSD methodology in empirical field studies with stakeholders as an attempt to understand their lived and embodied experiences of values of care. As noted in the introduction, we position field studies as a phase of research-for-creation in the wider research-creation approach (Chapman and Sawchuk, 2012). Research-for-creation involves gathering materials to inspire generation. Gathering can take many shapes, including scientific research activities such as qualitative and ethnographic methods. In field studies (see Section 3.1, we have focused on the expression of the CCVSD components context, care practice, actors (care workers and care recipients), and values of care, and have developed semi-structured interviews and card workshops based on these components. Instead of following the care ethic’s theory (Tronto, 1993) underlying the CCVSD, we adopt an inductive approach to gather values of good care from participants, acknowledging their lived experiences might differ from pre-conceptualized categories.

### 2.3 Speculative design approaches

As a second step to the research-creation approach, we have conducted a creation-for-research phase (Chapman and Sawchuk, 2012). Creation-as-research builds upon the inspirations gathered in research-for-creation phases–in our case the results from empirical studies following the CCVSD methodology–and follows a process of creative production aiming to communicate and reveal new forms of knowledge produced outcomes.

Methods from similar traditions are well established in HCI (Forlano and Halpern, 2023) and are starting to be more frequently used in HRI (Luria et al., 2021). According to Blythe (2014), design fictions can express novel perspectives or conflicts through speculation. The format remains ambiguous to solving the conflicts, while an emerging discussion of the fiction can lead to potential answers (Blythe, 2014).

Several studies in HRI have implemented speculative and fictional work as part of their inquiry (Auger, 2014; Cheon and Su, 2018; Dörrenbächer et al., 2020; Luria et al., 2020; Albers et al., 2022). Dörrenbächer et al. (2020) worked with student-created video design fictions of social robots as starting points for a secondary analysis. As an outcome, the authors discussed a variety of imagined application scenarios, social roles, and the benefits and challenges of social robots entering everyday life situations. Designing for informal care, Albers et al. (2022) initially interviewed informal caregivers and care recipients. Based on identified practices and a concept of “robotic superpowers”, the researchers developed speculative video prototypes of robots as means to explore fictional everyday situations together with participants.

In an interview study with roboticists, (Cheon and Su, 2018), co-developed “fictional autobiographies” of robotic concepts. The aim was to elicit the values of participants from created narratives. (Auger, 2014). investigated why robotic technologies did not yet become part of everyday life. He argues that approaches such as speculative design can help developers to focus on the everyday lives of people and less on technology-driven solutions. Similar to these approaches, our application of speculative design is grounded in analysis of the care context (Albers et al., 2022), without aiming to provide technological design solutions (Blythe, 2014), but to initiate discussion about values in care (Cheon and Su, 2018) and think through the influence robots exert on these in everyday care (Auger, 2014).

## 3 Methodology and methods

We frame the activities and outcomes of our studies as a research-creation (Chapman and Sawchuk, 2012) practice. As research-for-creation we conducted field studies (ethnographic observations, interviews, workshops) to understand the everyday lived experiences, circumstances, and desires in care. Following the CCVSD methodology (van Wynsberghe, 2013) we aimed to understand embodied meanings of good care and how values structure everyday care practices. As a practice of creation-as-research (Chapman and Sawchuk, 2012) we have written a set of speculative vignettes. They function as a rhetorical tool to express context complexities and tensions arising when we introduce hypothetical robotic technologies in care situations.

We approach our work from a constructivist epistemological paradigm, employing qualitative methods. Knowledge from this standpoint is actively constructed and contingent of knower and the circumstances. As researchers, we are actively involved in the construction of knowledge and strive to produce more informed understandings of the world (Guba and Lincoln, 1994).

All research activities and the informed consent procedure were submitted to and peer-reviewed by the research ethics committee of one of the involved universities and the ethics committee of the care organization. All participants received and signed informed consent forms and data processing agreements prior to participation.

### 3.1 Field studies

The first step of our research process was divided in two phases. First, we conducted observations and interviews, which is a common approach in PD and VSD and useful to explore and understand the context a robot is designed for (Moharana et al., 2019; Ostrowski et al., 2021). In a second phase, we developed a card tool along the CCVSD methodology (van Wynsberghe, 2013) to discuss practices of good care and conducted three card workshops. We conducted six interviews with three care workers in the care home and three care workers in mobile care, and conducted 3 card workshops that each involved one care recipient (n = 3) and one care worker (n = 3). In total, 10 unique individuals participated in our study, since two care workers participated in both an interview and a card workshops.

To acknowledge the co-constructive nature of knowledge production in our approach, we provide accounts of positionality, or information on the participants and researchers involved in this study (Reich, 2021) to promote the transferability (Korstjens and Moser, 2017) of results. Studies took place in a mobile care context and a long-term care home in Austria, both services offered by our research partner institution. Card workshops were exclusively conducted in the care home due to organizational constraints. In Austria, mobile care involves delivering care services to individuals in their own homes, with care workers traveling between locations. In contrast, a long-term care home is an institution where residents permanently reside. As explained by our practice partners and participants, in mobile care a broader range of people receives care, while in care homes the main group of care recipients are older adults and people with dementia. Care in both contexts includes support for Activities of Daily Living (ADL) (Katz, 1983), such as wound care, medication administration, or bathing, as well as Instrumental Activities of Daily Living (IADL) (Katz, 1983), such as meal preparation and household tasks. In Austrian healthcare, care professionals are differentiated by their level of competences and responsibilities, based on the underlying training. Participants covered the whole range of care qualification levels: i) qualified nurse (Bachelor degree level); ii) qualified care assistant (2 years training), iii) care assistant (1 year training), and iv) home assistant (1 year training). In Austrian healthcare, large numbers of care workers have immigrated from another country. All participating CWs had an immigration history, mostly from countries Southeast Europe, which is representative for both care contexts.

The authors are a group of interdisciplinary researchers collaborating on a project of designing robotic technologies for good care in participatory design approaches. Our scientific backgrounds are varied and include Human-Computer Interaction, Human-Robot Interaction, Cognitive Sciences and Science and Technology Studies, Sociology, and Media Design, hence none of us has experience of directly working in healthcare contexts. We were aware of our unfamiliarity and foreignness to care contexts ahead of the empirical work, which reflected our choice to first develop a nuanced understanding of the practices, actors and values.

#### 3.1.1 Observations and interviews

Seven observation rounds were conducted directly prior to the interviews by the first (3 observations; care home), third (1; care home), and fourth (3; mobile care) author. We accompanied 7 CWs on their working day, each for 4–8 h. Researchers followed an observer-as-participant (Flick, 2009) approach, as they were overt in the field but did not actively participate in the practices. Observers took field notes on actors, time, spaces, care practices, artifacts, and interactions. Based on immediately these notes, we wrote textual observation protocols, however, we did not analyze observation data. Our motivation to conduct observations was: a) To establish trust and familiarity between participants and interviewing researchers (6 of the CW participated in following interviews) and increase the likelihood of high information power (Malterud et al., 2016), b) to use observed situations of care as specific examples and discussion points in interviews, c) to gather concrete actors, spaces, and practices of care and adjust the card workshops to the actual circumstances of participants, d) to observe the procedure of care practices over a full day to inform the creation of varying speculative vignettes.

We conducted six semi-structured interviews (50–90 min) with CWs to explore their subjective understandings of good care. The interviews covered CWs’ experiences, opinions, thoughts, and feelings of care workers regarding the challenges of providing good care. All interviews were audio recorded and transcribed. Participants were care workers working in mobile care (n = 3, all female) and a care home (n = 3, all female). Interviews were conducted in German, the primary language of the interviewers and the secondary or tertiary language of the interviewees.

#### 3.1.2 Card workshops

Following the interviews, we noticed that CWs would often initially define good care as dependent on the person receiving care, and on the particular practice in question. Given this bi-directional, relational nature of care practices (Bjornsdottir, 2018) we recognized a need to discuss interpretations of good care with CRs and CWs together. For this purpose, we developed a card workshop method. Card workshops are a versatile method used in PD (e.g., (Beck et al., 2008)) and VSD (e.g., (Friedman and Hendry, 2012)) as they are an accessible material to facilitate stakeholder participation (Beck et al., 2008) and discussion on abstract issues related to places and practices (Schwaninger et al., 2021))such as good care. We operationalized the care-centred framework (van Wynsberghe, 2013) as a card tool to discuss good care with CWs and CRs. Given the scope of this paper, we do not offer a detailed description of the development and application of the card tool here.

We held three workshops at the care home where we had conducted our field studies, each lasting 57–78 min. Each workshop involved a CW and a CR. CWs (aged 24–55 years, all female) with varying levels of qualification participated. Two of them took part in the observations and interviews beforehand. Our practice partner facilitated recruitment of CRs (all above 65 years, all female) station leads consulted available Mini-Mental State Test (Folstein et al., 1975) scores, for residents and their personal assessment, as the workshop was not designed to accommodate people with moderate or severe dementia.

### 3.2 Data analysis field studies

We performed a reflexive thematic analysis (RTA) according to guidelines by Braun and Clarke (2022) to analyze the data from the interviews and workshops. They emphasize that RTA aims to generate rich, contextualized meanings based on the researcher’s deep, iterative, and interpretative engagement with the data (Braun and Clarke, 2022). With our goal to develop themes as everyday meanings of good care, RTA was a suitable method for data analysis.

The concept of information power as an indicator for the richness of a dataset guided the composition and size of our sample (Malterud et al., 2016; Braun and Clarke, 2022). We continuously reflected on our dataset and adjusted the data collection accordingly. We strove for a high specificity and variability (Malterud et al., 2016) in our sample to develop rich and diverse interpretations of good care. Our sample included seven unique CWs with varying professional expertise (assistant to leading positions; medical to social care foci) and experience (a few years to multiple decades) and 3 CRs with diverse reasons for being in care (sarcopenia, terminal illness and depression, and stroke-induced hemiplegia). Given the composition of our sample, however, we acknowledge that the interpretations of good care developed in our analysis capture primarily CWs’ perspectives. Additionally, after each workshop and interview, we reflected on the quality of dialogues and observed a relaxed atmosphere between researchers and participants, participants as open to talk about sensitive and personal aspects of care and comfortable to be challenged about these notions. Our reflections indicated a high quality of dialogue, which is an indicator for high information power, and thus justifies a smaller sample (Malterud et al., 2016).

The first author performed the RTA in regular conversations with the other authors. Particularly, the first and second authors had daily calls throughout the process of analysis to reflect on the progress, discuss challenges and reflect on the developed codes. Following Braun and Clarke (2022), a first step involved familiarization with the data, which the first author did by marking relevant sections in transcripts while listening to audio recordings in MAXQDA^1^. In the second step, the first author revisited marked sections and iteratively coded them. The analytic focus of the coding was on how participants constructed interpretations of good care, and which aspects were involved in these interpretations. After multiple rounds of coding, he examined codes with a larger number of instances for granularity of meanings and split them into multiple new codes if necessary. The first author presented these later versions of codes and underlying data extracts to all authors towards the end of the coding process. The first author developed multiple versions of themes during these discussions, which were drawn in a paper notebook. Clusters of codes were then replicated on an online whiteboard. By revisiting the codes and data extracts, the first author developed organizing concepts for the themes and explored the structure of and relationship between themes by creating multiple versions of slightly altered clusters of codes. A final theme had to be organized around one single coherent concept of good care of considerable depth and diversity, and simultaneously be distinctive from other themes. We wrote up our final themes based on the clustered codes, and searched and added illustrative transcript excerpts. We translated excerpts from German to English, with attention to making as few edits as possible for readability while staying true to the intended meaning of participants.

### 3.3 Speculative vignettes

The second part of our study resembles a practice of creation-as-research (Chapman and Sawchuk, 2012). Our goal was to create formats of design fiction, opening a space for discourse and consideration without proposing design solutions (Blythe, 2014) in response to RQ2 (”“What should we consider in the design of robots based on identified meanings of good care?”). With this, we mean that our paper mainly contributes to HRI research by opening the discourse around the introduction of robotic technologies in care. By placing a hypothetical, yet not impossible, robot in such moments, and articulating the possible consequences on care values and tensions, we contribute to the questions what we should consider when introducing robotic technologies. To this end, we collaboratively developed six speculative vignettes addressing some of the tensions that surfaced in our analysis.

Primarily used in sociological and anthropological research, vignettes have two applications. First, as representing a snapshot of lived experiences gathered in field studies. Second, as a short fictional scenario introducing characters and situations to elicit responses from participants (Spalding and Phillips, 2007; Jenkins et al., 2016). As a speculative format, they can explore how everyday conditions might be affected by introduction of technologies (Frauenberger, 2021). We identified vignettes as a promising format for design fictions articulating speculative scenarios of robotic technologies in care based on actual field study data.

All authors were involved in drafting or iterating these vignettes. We followed a step-by-step guide for generating vignettes, starting with reading the themes of our RTA. As part of our field studies, the first, third, and fourth author have conducted observer-as-participant observations (Flick, 2009) in both care contexts. Observation notes were made available to all authors in the vignette creation process. This ensured vignettes would consider sensitivities to the practices, procedures and actors in care situations, crafting scenes close to those in real-life. Following reading themes and observation notes, each author chose one robotic concept from a selection conceived at an internal brainstorming workshop. Authors first individually wrote vignettes, which were later refined in a collaborative and iterative writing process, ensuring that illustrated scenarios and envisioned technologies remained open for interpretation and expressed a tension observed in our analysis. The first author made final edits, such as placing all vignettes in the course of a day in a care home, given he was most familiar with this context, and to aid the reader in imagining the vignette scenarios through recurring locations and actors.

## 4 Results

In response to RQ1, we present five themes as results of our RTA process. In Table 1 we provide an overview of the themes with the corresponding core organizing concepts.

**TABLE 1:** Overview of themes and organizing concepts.

	Theme	Summary
(1)	*Building a Trustful Care Relationship through Time and Routines*	Trusted care relations are built in processes considerate of time and consistency in care routines
(2)	*Negotiations of Care between Care Stakeholders*	Ongoing negotiations of care stakeholders (CWs, CRs, relatives) shape the performance of care practices and require balancing of multiple perspectives what good care entails
(3)	*Emotional Care and Reciprocity*	Performing care practices well involves creating spaces for CRs to express physical and emotional vulnerabilities. CWs in turn draw emotional validation from attending to these vulnerabilities
(4)	*Self-Care of Care Workers*	In order to provide good care, CWs work in self-caring practices and attitudes
(5)	*Socio-Material Mediation of Care*	The socio-material circumstances of care mediate performance of good care, illustrated in information exchanges, care planning and lifting practices

### 4.1 Theme 1: Building a trustful care relationship through time and routines

The first theme is organized around the processes of building a care worker-care recipient relationship. Two facets illustrate how a trusted care relation is established through the performance of a consistent care routine, with consideration of the time required to build routines and relations.

#### 4.1.1 Consistent care routines and relations

The consistent execution and collaborative development of care routines are crucial for providing good care, as they foster trust and familiarity. Ideally, the CR-CW relation is maintained by having only a small group of care workers performing care with a group of care recipients. Both CW and CR can form expectations for the performance of care. *“Well, we are doing patient-centered nursing, that is, we try as much as possible that the same care workers always go to the same clients, so their relationship is better established.” (Mobile Care Worker 1 = MCW1)* This is especially important for people with dementia. Any disruption in established routines or unfamiliarity with a CW could cause confusion and resistance against performing care practices. *“Especially with clients with dementia, patient-centered care would be important, where you know the processes are in place.” (MCW2*).

In essence, a consistent care worker-care recipient relationship and repeated performance of a routine transforms the whole care process from initial novelty into one characterized by familiarity, supporting the expectations and trust of CR in CW. *“And then they are happy when I come, because they already know me. And then I also do not have to always repeat, you do that, you do that. Because they already know and they trust us.” (Care Home Care Worker 1 = CHCW1)* Consistency eliminates the need for a continuous explanation of the practices, which CWs in turn, interpreted as signs of trust and familiarity. A CR supported that familiarity with CWs and experiences of consistent, proficient, and repeated performance of care practices reduces their fear and, in this sense, expresses a form of trust. *“Actually, my fear is taken away, when I see there is a care worker and another one, anyway two that I know and I know I can rely on them.” (Care Recipient 1 = CR1*).

#### 4.1.2 Taking time to build routines and relations

Particularly in unfamiliar care circumstances, developing care routines requires time. Time is needed to allow for a collaborative development of routines involving identification of and adaption to preferences and habits of CRs. For instance, this might involve performing the same care routine at a specific time each morning. *“Customers are accustomed every day, almost at the same time, that someone comes, washes her, dresses her, prepares breakfast, prepares medication.” (MCW1)* CWs acknowledged making only minor changes at first to ease the transition into new routines and being understanding of the time it takes for a CR to become used to them. As indicated in the first facet, new routines but also new CW-CR relations require a period of acclimatization. *“Time plays the big role, yes. To learn how to interact with the person. They also learn (to interact) with us.” (CHCW2) “I am there more often. She (the CR) probably will not find it so difficult when she cannot do it anymore and we decide I will wash her back or something. Doing the little things first. And when (a) completely new (CW) comes people need a settling-in period.” (MCW2)* As the care routine slowly becomes established through small changes, the interpersonal space and trust between CW and CR start to grow, allowing for conducting increasingly intimate physical care practices and more emotional care, as will be explored in theme 3. *“She knows me, so it probably will not be so difficult if she cannot do what she can now. Where I then said, well, I can wash your back or so. First allowing little things. And when someone completely new [comes] the people need a period of acclimatization.” (MCW2*).

It is essential to mention that continuity in care relations, consistent routines, time for routine development, and building trust through routines are interconnected, forming a holistic process in developing care relationships.

### 4.2 Theme 2: Negotiations of care between care stakeholders

Our second theme delves into the collective nature of shaping care. Our data included the ongoing negotiations of CWs, CRs, and relatives shaping the specific performance of care practices. While other actors undoubtedly contribute to these negotiations, our reporting focuses on negotiations between CWs and CRs, and CWs and relatives.

#### 4.2.1 Care worker - Care recipient negotiations

In the first facet, we report aspects of the negotiations between CWs and CRs. The interplay between the autonomy and independence of CRs and the imperative for CWs to nurture their wellbeing requires a delicate balance. One way of CWs to preserve the independence is by sustaining their existing abilities without further diminishing them. To illustrate, in mobile care this could entail dividing household tasks in a manner that accommodates a CR’s capabilities on a given day. They may participate in washing the dishes, drying them, or doing both, or walking as far as they are able and being pushed in a wheelchair for the remaining distance. As the capabilities and preferences of CRs change over time, the distribution of participation requires continued and repeated negotiation.

The autonomy of CRs extends beyond deciding the performance of physical activities, as it encompasses a general freedom of choice. CWs expressed a commitment to acknowledging the wishes of CRs and the individuality coloring these preferences, as illustrated in an extract on the duration of staying in the garden for tea time: *“Some people do not want to stay out that long. But that’s also something. Fifteen minutes, half an hour. It’s very individual. Everybody is different.” (CHCW3*).

In this sense, eliciting articulations of immediate needs was pivotal in practices. CWs reported constantly addressing CRs to express their needs, preferences, or choices. Conversely, CRs highly value the capacity to make choices and have them respected by CWs. *“You know what is very good? The care workers come and if I do not have pain I go with them (to the garden) and if I have pain I stay in my room. And that’s okay. They say it is good, it is okay. And I think it is right that you’re not forced to come along.” (CHCW3*).

Challenges arise when CRs lose the ability to effectively express themselves, such as in advanced stages of dementia. CWs face difficulties in interpreting observations instead of relying on verbal expressions. This can introduce tensions, as the interpretations may be inaccurate. In such situations, a close relationship, a deep understanding and collaboratively established routines become valuable for providing good care along (assumed) preferences of CRs. *“We do not know anything about the residents and then we do what we suspect is best, but maybe that is not right.” (CHCW1) “If I know who likes what, and who dislikes what, then it is easier. If not, then I have to guess. And that all comes from experience, from that you get to know the people.” (CHCW2*).

CWs work from a care plan that prescribes the care practices that should be performed for a CR. Another tension arises when there is a mismatch between the preferences of CRs and CWs’ aspiration to perform “all” care practices of the plan. *“And it is important to me that I do all my professional, caring measures. That they get the treatment which meets all their needs. I’m happy when I get my work done. From A to Z, yes.” (CHCW2)* CWs perceived it was their responsibility to balance caring according to the care plan while continuing to consider the autonomy of CRs in making choices for their care. Finding this balance becomes increasingly difficult if CRs cannot express themselves directly, and it becomes unclear what their choices are. One approach to handle such situations was for CWs to motivate CRs for a care practice, even if it might contradict their initial expression of preference, such as in the following excerpt referring to a person with dementia who refused to eat. *“And she says: Well, I do not want to eat. You can motivate. Try at least a spoon or two, or at least something to drink.” (MCW3*).

#### 4.2.2 Care worker negotiations with relatives

The second facet of this theme related to the dynamics and tensions between CWs and relatives of CRs. On the one hand, CWs build and maintain relationships with relatives, especially those living with CRs in mobile care. Care includes the needs of relatives, such as temporary relief from the burdens of care, or offering emotionally loaded care, such as end-of-life care. When CRs cannot express themselves, relatives become a crucial and often only source of information. *“If the resident is in a palliative stage, then we as care staff - we also care for relatives, so they help us at the beginning and then we help them.” (CHCW4*).

On the other hand, reliance on relatives can lead to ambiguities in performing good care, for example, when relatives lack up-to-date knowledge of the CR’s (current) preferences, habits, and needs or project their assumptions on the CR’s situation. Not only are the voices of CRs diminished in such moments, but CWs find their approaches to care challenged. Relatives set unattainable expectations of the quality, immediacy, availability, and “correct” care performance. CWs must navigate these circumstances by working from their expertise and capacities while prioritizing the needs of CRs and, simultaneously, avoiding conflicts with relatives that could threaten their care partnership. *“Often the ideas of the relatives, are different, to what the client wants.” (MCW2)* One example of a CW navigating their available capacities with the unattainable expectations of relatives is illustrated in the following excerpt. *“I’m trying to change something. If several times it is not okay, then it is no longer my problem. I can not do it on the millimeter, it is not possible in care to make everything perfect. If they complain I try to talk back nice. (I tell them) I will try to change it.” (CHCW4)* The CW initially showed readiness to adapt the care practice according to the suggestions of relatives but would stop with repeated and increasingly meticulous demands. Despite a lack of understanding of the CWs’ capacities, CWs remained amicable to avoid strains in the care partnership.

### 4.3 Theme 3: Emotional care and reciprocity

The third theme depicts how performing care practices involves creating space for and attending to the physical and emotional vulnerability of CRs and how CWs draw emotional validation from attending to these vulnerabilities in acts of reciprocity.

#### 4.3.1 Opening spaces to allow vulnerabilities in care

In developing a trusting, routine-based relationship (as discussed in theme 1), more spaces for disclosure of vulnerabilities open up. In an illustrative example, a CW described how a CR would only accept one particular CW for wound care. *“Then we have a customer, where only I go. Because she just has the trust in me. She’s ashamed of the wound that she has. And she does not want a change of staff.” (MCW1)* This example illustrates an (accelerated) process of building a trusted relationship through a care routine, which allowed the CR to accept and CWs to attend to their vulnerabilities. Further, the example reveals an interconnectedness of attending to bodily and emotional care.

In the example above, emotional vulnerabilities are cared for implicitly as part of a bodily care practice. However, at other times, CWs explicitly address the emotional care needs of CRs. This may involve actively inquiring about the emotional wellbeing of CRs and fostering opportunities for them to share their feelings as part of their trusted relationship. Many CRs would rarely have the chance to do so, e.g., due to reduced social contacts, and would take the opportunity to discuss sensitive concerns such as end-of-life thoughts or feelings of depression. *“She was really sad and when I was there, but usually she always tries to be cheerful. And then I asked her, ‘What’s wrong with you today, tell me.’ And then I gave her time and asked, do you want to talk about it. And then she said but what I tell you, please keep it between us.” (MCW3*).

Directly inquiring about emotions was viewed as a unique care practice, a pivotal opportunity to build and strengthen care relationships. In these one-on-one conversations, care workers function as listeners and active participants, offering personal advice and emotional support. Despite their significance, these practices are often undervalued in care planning and discussions about quality care, according to CWs.

Engaging in such practices allowed CWs to develop a heightened ability to recognize and interpret expressions (speech, gestures, mimic) as signifiers in care interactions. This becomes important again for CRs who may have limited or impaired verbal communication, such as people with dementia. *“Some tell you, but some do not. For some it is not clear what they want. We can not understand, but we must watch closely. And after a longer time we know better what they want and what they do not want. How is the facial expression, you can also know from that.” (CHCW1*).

Through interactions depicted above, closer bonds develop between particular CRs and CWs, often ending in CRs favoring certain CWs. The factors influencing these preferences are unclear, with CWs attributing them to a distinct interpersonal chemistry. *“And there is always one person who the resident fixates on. And that does not mean that others cannot do the care. But just that this person has the most trust from the resident.” (CHCW2*).

#### 4.3.2 Care workers experiencing emotional reciprocity

While the first facet of the theme was centered on the emotional experiences of CRs and how CWs attend to them, the second facet concerns what CWs experience regarding emotions, support, gratitude, and fulfillment for their care efforts. Care workers described emotional capabilities as a central quality of their professional role, most often depicted as having an open heart and working from a stance of compassion. As illustrated in the following extract, working from compassion allows CWs to help other people, starting with identifying and understanding care needs. *“You need to have a heart in this profession. Heart, patience, love, being there. We do not work just for money. We help and support other people as much as we can.” (MCW3*).

On the one hand, CWs reported experiencing increasing the wellbeing of CRs as a form of validation for themselves. *“And that makes me really happy when I can help, when I can do everything well then I go home with a good feeling. The resident is satisfied, we are satisfied and then everything is fine.” (CHCW2)* On the other hand, perceiving the thankfulness of CRs and their relatives was another confirmation that CWs are performing care well. *“A certain gratitude helps me to I realize through my work I can actually make people happier.” (MCW2)* We can identify a reciprocal quality of good care in that the act of providing care itself is valuable, but what CWs receive back in terms of emotional validation from CR is a contribution to a picture of good care. Overall, responsiveness to the correct performance of care was essential for CWs to perceive work satisfaction and self-identification with their profession.

The responsiveness from CRs to the care practice has a dual function for CWs. On the one hand, it confirms to CWs the ‘correct’ (in the sense of fulfilling needs and increasing wellbeing) execution of care practices. On the other hand, receiving confirmation for a central aspect of their professional identity positively contributes to the self-identification of CWs with their profession, and confirms their choice of profession. *“That gives me the confirmation that I’m doing the right thing that I’m trained for and that I’m in the right profession. It gives me pleasure, yes. Yes, when someone confirms that you’re doing well, it is a great joy.” (CHCW2*).

### 4.4 Theme 4: Self-care of care workers

While the first themes deal with the relational, collaborative and reciprocal elements of care, the fourth theme concerns an intra-personal dimension of good care. CWs described a number of practices and attitudes that we subsume under the notion of self-care. They report performing self-care practices as a means to provide good care for others.

In general, practices of self-care concern finding a balance between the responsibility of caregiving and related physical and emotional burdens and the responsibility for one’s own health and wellbeing. Care workers expressed awareness that qualitative care is only possible when they care for themselves. *“Trying to do a lot for yourself, take time for yourself. So that we have more energy, because for this profession you really need a lot of [mental/emotional] strength.” (CHCW3*).

Some self-care practices pertain to the direct performance of care: taking time, working with patience, and being in the present moment. On the one hand, being present in the moment was aligned with a self-image of good care and providing care according to the expectations of good care. *“For me it is really important that I have time for a customer, I’m present with the customer. This time belongs to them. They pay for it and I do not have to be mentally somewhere else.” (MCW3) “If you take time it is great for you and it is also great for residents.” (CHCW1)* CRs reported sensing when a CW was working under time pressure, connecting to elements of responsiveness and reciprocity, and pointing to the importance of having and taking time as a CW to perform good care. *“I can sense when a staff member is stressed, because then they’re quick. Then I know, then I already say, ah today you have a lot to do again.” (CR2*).

Certainly, in the face of staff shortages and underfunding of care services, working with patience is not always possible. Nonetheless, CWs indicated they own a share in ensuring they work from a stance of patience. *“If I do not have patience, if I’m constantly stressed, I’d better go and find something else (to work).” (CHCW3*).

Another facet of care worker self-care concerns a separation of private life and professional life. While affective care, as presented in the third theme, involved CWs establishing spaces of vulnerabilities for CRs, CWs, on purpose, withdraw their personal vulnerabilities from such spaces. Therefore, care is not fully reciprocal, as it embodies an inherently asymmetric relationship in which CWs do not expect care for their vulnerabilities. From a point of view, this can even be desirable for CWs, as it allows them to find distance to their personal burdens. *“Most of the time I come to work without thinking of my problems. Although I have a thousand problems outside I come here and everything is gone.” (CHCW4*).

CWs simultaneously created a boundary between professional concerns and their private life. This separation involved ending the sense of responsibility at the end of the working shift. As highlighted in the following excerpt, mastering this self-care measure is a professional competence that requires practice. *“I have somehow managed, when I go home, I just leave everything. I am completely somewhere else and I never call and ask what happened. Only when I’m [at the care home], I get all the information, but I do not take that home. It is easy to say and everybody thinks that’s so easy, but it is very hard to accomplish.” (CHCW3)* Our data only provided a narrative account of these practices, leaving us to speculate on how far the separation of private and professional life succeeded.

Last, self-care practices involve setting boundaries within the care work. CWs would reflect on their capacities and capabilities and refrain from performing care beyond these self-established boundaries. *“For me, I actually have my (…) limits. So I’m not the one who (…) does a lot beyond my limits.” (MCW2*).

Moreover, communicational boundaries for CRs aimed to maintain the CWs’ emotional/mental wellbeing. Care workers reported setting boundaries for CRs regarding communication style, for example, not accepting being sworn at. *“Even if we have screaming or hysterical clients now. I say in a calm tone, please, voice down. I will not let them yell at me otherwise I’ll terminate the visit.” (MCW2*).

### 4.5 Theme 5: Socio-material mediation of care

The final theme from our analysis makes the socio-materiality of care explicit, showing how materials-technologies, social structures, and care practices are mutually dependent and collectively contribute to a practice of good care. This became evident in three facets: information sharing, mediation of flexibility in caregiving, and safety-inducing materials for caring.

#### 4.5.1 Information exchange and documentation

Regarding information exchange, CWs described several practices for sharing information with each other. Two main types can be differentiated into verbal communication and transmission via digital systems. Technologies actively contribute to the exchange of information. Particularly in mobile care, CWs would share information via phone calls or group chats. For example, when a CR received a device, CWs would share photos and instructions with their colleagues. *“When I come to a customer and I see that she has a new [device], then I take a photo, send it to everyone.” (MCW3*).

Care workers reported a stronger emphasis on verbal transmission of information for care homes. In each shift, one CW would be on “main duty”, and collect all care-relevant information from other care workers in intermediary exchanges. *“The person on main duty must always be informed.” (CHCW1)* Verbal transmission was an immediate practice and relevant during shifts and at shift handovers. On the other hand, written reports were relevant as a legal obligation and from a future-oriented perspective. *“If you have not much time or someone forgets … then when you come back after a few days, you cannot remember. That’s why it is important to always document.” (CHCW2)* CWs need to make informed predictions and decisions anticipating potential future needs of care. This can include end-of-life care considerations or transitions between care contexts; hence, documenting this type of information in long-lasting storage, such as a care documentation system, has become important. *“Medical activities like life-prolonging measures such as resuscitation or artificial nutrition. Everything is written down so you can stick to it.” (MCW1*).

#### 4.5.2 Mediating flexibility of providing care

A second facet addresses how a digital time recording application mediates the flexibility of work practices. Writing documentation, including time recording of care visits, is a legal responsibility and is thus considered part of working hours. In mobile care, a digital time-keeping application is in use. In the following excerpt, a CW refers to a moment when they ended the care visit in the application before actually ending the visit. They continued to write the documentation as part of their travel time. *“But I still had the documentation to do. So I finished the visit [in the time recording application] but I continued. Because I did not want to exceed. Because when I start another 5 minutes then 15 minutes are counted. The customer pays 15 min. And then I realized that I do not even need 2 minutes to finish. And these 2 minutes I write as travel time.” (MCW3)*. Restrictions on time granularity and regulations for hourly fees drove the CW’s decision. Billing occurred only in quarters of an hour. The mediation of the application becomes evident when we imagine a different form of calculation and recording of care visits. Care workers generally desired greater flexibility in care planning within mobile care beyond the constraints imposed by current scheduling and regulations. *“That’s the disadvantage (…) I have 2 hours of visiting scheduled today. The weather is bad, so I would say I do half an hour today and tomorrow, when the weather is better, I’ll add the time and we go for a walk. Now the program wants to know why my visit was ended early today. I have to write it, but the assignment will be billed for 2 hours still. Even if I’m only there for half an hour. And the next day the client has to pay extra.”(MCW2)* Care workers reported feeling restricted in adapting the care planning to the immediate needs of CR and the daily circumstances, which stands in contrast with the desire to adapt care to the immediate needs of CR as reported in theme 2.

#### 4.5.3 Feeling safe through use of material infrastructure

The final facet of theme five revolves around care materials contributing to feeling safe while performing a care practice. For CWs in both care contexts two material infrastructures - hospital beds and semi-automatic lifting devices - contributed to their perception of performing care practices in safe manner. In mobile care, hospital beds (which are not available at every CR) allow CWs to work from health-sustaining ergonomic positions. *“Everyone should have a hospital bed. There are some people who do not have a hospital bed and some things are extremely strenuous, for example, when someone sleeps on the sofa. Imagine you have to do care there, bending down and so on. Your back will be worn out.” (MCW3*).

In a similar fashion, a lifting device was regarded as a contribution to a safe performance of lifting in the care home. Moreover, it would enable performance of a care practice, such as lifting a heavier person out of bed, in the first place. *“We’re lucky that we have a lifter at all, because without a lifter they (the CRs) can hurt themselves and we can hurt ourselves too. And you always have to mobilize when you are at work.” (CHCW4) “This lifter is really good, because some CR are impossible to mobilize even with fifty people. We absolutely need this lifter.” (CHCW2)* Both material infrastructures mediated essential care practices of lifting and turning. Having a technology mediate the practices to be safe and sustainable can be assumed to have a positive impact on the intention and perception of performing the practices.

## 5 Contributing design considerations through speculative vignettes

Contributing to RQ2 (*“What should we consider in the design of robots based on identified meanings of good care?”*), we present a collection of speculative vignettes as design fictions. The vignettes describe situations in care with robotic technologies, to which we provide considerations situated in our developed themes. In this sense, considerations address potential tensions but also spaces of possibilities for robot-mediated care. All vignettes have been created (see Sec. 3.3 for methodological details) to unfold within a day in a care home, akin to one visited during our field studies. We have organized the vignettes based on a temporal structure to address varying everyday care situations in a care home. We interrupt the temporal sequence with discussions of considerations.

The robotic applications, while fictional, represent technical capabilities that are feasible. The vignettes present applications such as a (robotic) alarm clock, robots with conversational capabilities, delivery robots (e.g., (Law et al., 2021)), and stress monitoring (e.g., (Samson and Koh, 2020)), These applications are mentioned here to illustrate potential tensions that arise when combined with the understanding of care that has been developed in Sec. 4.

Time: 06:15; Location: Mrs. M’s room

Like every day, at 06:15 the robot starts glowing in a smooth light simulating a gentle sunrise and waking Mrs. M up. When the robot detects Mrs. M is awake it sends signal to the care worker station. “A care worker will be with you shortly” the robot announces. “Who is it going to be? Maybe Nadine?” Mrs M responds hopefully. “Let me check...”

In theme 1, we identify the importance of collaboratively developing care routines. Thus, introducing robotic technologies in everyday care implies two considerations regarding routines. First, robotic technologies will enter existing routines and can influence the development of novel routines. In the vignette, a robot is waking a CR up. Here, the accuracy and predictability of technology–an inherent strength of technologies (Dörrenbächer et al., 2020)–could be used as an advantage in developing highly consistent routines, which was identified as a key characteristic of good routines in theme 1. Second, we need to consider the essential element of developing routines in that it allows CWs and CRs to establish and widen a trusted care relation. It is a design choice whether a robot facilitates the relation between a CW and CR, e.g., by sending message to the CW and by announcing to the CR a CW will shortly arrive, or if it does not.

A similar tension can arise regarding unique CW and CR relations, as articulated in theme 3. The CR in the vignette is asking for a specific CW. Indifferent from what the robot in the vignettes is doing in reaction to the CR’s request, we can formulate a third consideration in that robotic technology could be designed to support or inhibit one-on-one relations between CWs and CRs. What the robot may do in response to the request can depend on a number of factors: work schedules, workforce capacities, preferences of other CRs and the preferences of the CWs.

Time: 06:39; Location: Mrs. M’s room

“Can we perform a pain assessment routine for the arthritis in your fingers, Mrs. M? My care planning module indicates a new assessment is due today” comes from the robot just as Nadine and Mrs. M had finished the morning care. “Great timing” Nadine says to herself while thinking the opposite. She had already noticed the arthritis must have gotten worse, but she forgot the assessment was due today. The problem is, Mrs. M does not like to talk about it at all. Nadine can already notice the reaction in Mrs. M’s facial expression. “You know what, let’s skip the assessment today, shall we” Nadine quickly says. Visible relief sets in on Mrs. M’s side. “But I think it would be good if we can do some exercises for your hands together, what do you say?” “Fine,” comes back following a short moment of consideration from Mrs. M. Nadine skips the assessment, knowing very well it will reappear tomorrow, and searches for the arthritis relief exercises on the robot’s interface.

In our second vignette, we can identify how a highly accurate robotic technology for routine facilitation (theme 1) could create tension in the care relation rather than support it. Additionally, the vignette illustrates how a partially autonomous robot would enter existing negotiations within care processes (theme 2). The robot in our example embodies qualities CWs had expressed - it elicits preferences and information of CRs and desires to perform all care practices (i.e., the pain assessment). However, bringing the pain assessment up despite the CRs personal preferences to not talk about it, raises tensions and creates a space of choices that might not have emerged without the robot. We can assume a CW with an established relation to the CR, such as in the vignette, would not have addressed a sensitive topic in the same manner. As it has emerged, the situation needs resolution. In a productive attempt, the CW in the vignette uses the robot as a counterpart and dismisses it to find an entry point to motivate the CR to perform a practice beneficial to their wellbeing (i.e., the exercises). Just as the CW had grasped agency to resolve the situation and shifted the tensions, other actors could act and shift the scenario. To summarize, when designing robotic technologies for care, one needs to consider the negotiating practices of care and how the role of the technology in these negotiations is always an active one.

Time: 09:06; Location: Hallway

Jelena, the intern of the care station, is picking up the dishes from breakfast. She moves from room to room while the autonomous service cart trails behind her, following every footsteps but lingering in the corridor whenever she enters a room. Then the thing happens again. Sometimes the cart would not wait in front of the room she had just entered, but move ahead to the next room. No one on the floor really knows why it’s happening. They speculate it might be to make them work faster, but it’s almost happening randomly. Anyway, Jelena doesn’t like it when it does so. Another care worker told her to just turn the thing off every time she enters a room.

The third vignette touches upon matters of work process structuring (theme 5) and preferred working styles of CWs (theme 4). Our analysis shows that care infrastructures, including digital technologies, enter and mediate care processes (theme 5). In the case of the vignette, the cart exerts influence over the pace of collecting breakfast dishes. Contrary to a purely manual cart, the autonomous cart in the vignette actively dictates the CW to move through the rooms in a certain pattern, resembling the conditions around a time recording software described by our participants. Thus, one design consideration is to anticipate and assess the influence of robotic technologies over the organization of work processes in care.

In theme 4, we discussed how patience is integral to performing care practices. From this perspective, introducing a technology that sets a different pace to the routines may be negatively experienced by both caregivers and care receivers. Therefore, our second consideration emphasizes the importance of remaining attuned to particular ways in which technology “choreographs” (Coeckelbergh, 2020) and reshapes care routines, their content, and their temporalities. When this is not considered, it risks resulting in non-use, sabotage and/or altered subjective experiences of care practices that may counter the values and expectations of good care.

Time: 12:31; Location: Duty Room

Nadine takes a deep breath as she walks into the duty room. It’s the first break of the day, and the first moment she has to think about the fight she had with her partner the day before. The robotic unit on care worker “self-care” support has already detected an unusual stress level via sentiment analysis and biofeedback data during her working shift. At the same time as it processes whether to initiate a routine check-in dialogue in the back-end, a tear trickles down Nadine’s face.

As a second facet of self-care, we have identified CWs engaging in practices of separation between work and private life (theme 4). In principle, insights regarding self-care are valuable and can lead to increased attention to support the wellbeing of those providing care to sustain good care overall. In the vignette, a robotic technology performs assessments to detect harmful stress levels in CWs, which could be one role for a robot derived from calls for CW self-care support. However, as illustrated in our themes, self-care can mean pausing private problems during working hours. The robot in the vignette could break this barrier if it initiates the ‘check-in dialogue’ and augments the fractures in the CW’s attempt of self-caring separation as much as it could be positioned as an emotional outlet without stigmatization based on the content (Breazeal, 2011). Therefore, in case robots enter practices of CW self-care, we have to consider CWs’ existing practices, desired constitution, and nuanced individual choices in relation to them.

Time: 16:18; Location: Mr. G’s room

As usually in the afternoon, Mr. G is sitting at the table in his room, waiting for tea to be served. For a few weeks these new carts have been serving it. On the minute, the cart enters Mr. G’s room. “Good afternoon Mr. G, today I bring you herbal tea and a brioche” the cart announces as every day. “Ah, brioche” “Do you like brioche? For any particular reason?” the cart goes on unexpectedly for Mr. G, as it detects another answer than just a “thank you”.

In the above vignette, a scene from an ongoing process of relationship building between a CR and the robot can be observed. We draw connections to theme one and how developing care routines and relations requires sufficient time and incremental expansions of interpersonal spaces. In the vignette, the cart has been serving tea for “a few weeks”, but to the surprise of the CR, it asks him a follow-up question. We can imagine Mr. G’s bewilderment if the cart had been doing so the first time it had ever served tea autonomously. However, we are left to speculate how Mr. G reacts to the cart, given that it has never interacted further with him. We emphasize developing and introducing robotic technologies to care contexts in incremental and relationally sensitive manners. We may deal with a concept similar to the novelty effect (e.g., Smedegaard, 2019; Fraune et al., 2022). Instead of developing a solution that faces an initial surge of interaction before a sharp decline, developing relations with robotic technologies may benefit from slowly but steadily increasing the complexity of interactions and thereby lead to more sustainable use.

Furthermore, the vignette leaves unanswered whether the CR even wants to interact with a cart beyond having tea delivered. Technological capabilities should not impose requirements to use them, i.e., just because the cart can hold dialogue does not mean the CR has to engage in it. We formulate this design consideration in line with theme 2, expressing the importance of eliciting and following CRs preferences to the performance of care practices.

Assuming they would engage in a practice eliciting personal information from CRs, we should consider how CWs can benefit from such interactions. As theme five pointed out, information exchange and documentation are performed in varying practices, with an essential point being that relevant information for care is made available to care stakeholders across time. Therefore, in designing robotic technologies with capabilities to engage, record, and process conversations with CRs, we should consider if and how this information can be curated for CWs. Moreover, we should consider the autonomy of CRs in these instances, considering their say in which information is stored, who might have access to it, and what should not be documented.

Time: 20:52; Location: Duty Room

“The biography sheet suggests it is Mrs. O’s preferred bed time soon” the robot informs Maria. So she gets on the way to Mrs. O, the most recently admitted CR. O is living in a rather advanced stage of dementia. Unfortunately, that’s almost everything they really know about her. At night, she is often irritated, crying and wandering a lot. The CWs are still figuring out how the bed routine can be adapted to her needs. In that sense, the robot wasn’t too helpful as it just gave an average of what O’s children filled in via the digital biography sheet - and for preferred bedtime, they gave contradicting information.

In theme 2, we interpreted the interactions between CWs and relatives of CRs as another type of negotiation influencing specific performances of care practices. Like in the reporting, the vignette describes a situation in which information from relatives is sought after, as the CR cannot express certain information anymore. However, the contradicting nature of the information places doubt on what is accurate for the CR. Again, the robot enters these negotiations. This time, the technology computationally translates the information, amplifying some aspects (i.e., building the average) while reducing other dimensions (i.e., the contradiction). One of the suggested bedtimes might be preferable for Mrs. O. However, if the technology conceals it, it may take longer to identify which is the correct one. Again, we must pay consideration to the specific shapes a technology enters negotiations of care performances. Thinking along the lines of the unique capabilities of humans and robots and how they can be productively interweaved can be a useful approach for conceptualizing them (Dörrenbächer et al., 2020; Albers et al., 2022).

## 6 Limitations and future work

Our qualitative studies were conducted in Austrian care home and mobile care contexts. We acknowledge the diversity of care circumstances, actors and practices, and therefore cannot claim to represent all possible perceptions and interpretations of good care in Austria or elsewhere. What can be perceived as another limitation, is the number of participants (n = 10) in our study. We point out that the perspectives of CWs shaped our interpretations to a larger extent than the perspectives of CRs. This inclination was due to pragmatic considerations in recruitment and research capacity. However, working from a constructivist paradigm and employing qualitative methods is incompatible with claims to generalizability, but should be judged upon the transferability of results to other contexts. Facilitating conditions for transferability are a provision of contextualizing information and providing “thick descriptions” of qualitative findings (Korstjens and Moser, 2017). We argue that the 10 participants in total yielded a rich set of perspectives to draw from. They covered different positions (care workers, care recipients, care homes, mobile care) and were engaged in different ways (through interviews and card workshops). We have captured their tacit, embodied, and enacted understandings of good care in five densely described themes. We believe they are transferable with regard to the (good) care principles they bring forth and the tensions they highlight involved in the introduction of technologies into care context. That said, our own data collection and analysis could benefit from more diverse groups of participants, including more CWs of varying qualification levels and from different care contexts, and particularly including more CRs. For future work, we encourage robot designers and developers to construct a deep understanding of the particular and lived conditions under study. We hope our work can provide guidance and reference in developing understandings of good care through noticing similarities and differences to the values identified herein.

In this paper, there is limited consideration of technological capabilities, however, the focus of our work was on developing a better understanding of the care context and understandings of good care in Austria. We demonstrate the relevance of this better understanding of care to the development of robots by illustrating tensions that inevitably arise through speculative vignettes. Technological capabilities assumed in the vignettes are not infeasible, as evidenced through reference to comparable systems in development and deployment. The transferability of discussions of vignette scenarios to other care contexts lie not in the imagined systems, but in the relations between care actors, systems and care values.

Nonetheless, the exact technological components and functionalities of a system will exert influence on the effect of systems on inherent tensions of good care. Moreover, the perceptions, values and beliefs of stakeholders towards a robotic systems will shape the acceptance and use. Therefore, we point out two takeaways for further steps in developing concrete technological solutions. First, participants should be included in design processes to integrate their assumptions and values. A key area to successfully integrate robotic technologies in care is re-conceptualizing the role of stakeholders (Frennert and Östlund, 2014) and involving and empowering them to have a direct choice in design. Stakeholders in the present paper had the role of informants. In a next step, they could be invited to discuss researcher-created vignettes to express their concerns and opinions directly. Genuine formats of active participation in HRI have been called for (Lee et al., 2017; Weiss and Spiel, 2022) and have started to find their way into practice (e.g., Moharana et al., 2019; Ostrowski et al., 2021; Winkle et al., 2021) pointing to fruitful future studies.

Second, developing robotic solutions from the identified tensions and in participatory formats has to build bridges to available technological capabilities. We suggest future studies to understand the themes and tensions of good care as a guiding tool in developing solutions. Our practice of informed speculation through vignettes is transferable to other situations in which designers and developers build robots. Knowing the functional capabilities and intended use cases, developers can envision their concepts in informed, hypothetical scenarios, before making larger investments into prototypes. Thinking through the scenarios with an understanding of the values of stakeholders - either indirectly through qualitative accounts or directly in participatory formats, can illuminate promising and frictional pathways early on in the development process.

## 7 Conclusion

In conclusion, our study offers an approach to identify values and tensions in care contexts, and how technologies will enter these circumstances. We have conducted interviews and card workshops in a care home and a mobile care context to develop a reflexive thematic analysis. The results of our analysis contributed nuanced insights into the situated and embodied practices of care and the interpretations of good care and respective values. Our themes illustrate the importance of mutually developed care routines, balancing of ongoing negotiations between care stakeholders to performance of care, affective care spaces as possibilities to express and receive care for vulnerabilities, the reciprocal qualities CWs draw from caregiving, effective provision of care rooted in self-care of CWs, and the ongoing socio-material mediation of care performances. Furthermore, we developed six speculative vignettes as formats of design fiction. The strength of our work lies in discussing considerations and tensions that arise in developing and integrating robotic care technologies for care with developed themes.

We believe our work is valuable to a broader audience working on technological solutions to care contexts and other sensitive fields. Our contribution lies in illustrating how we can bridge the contextualized and situated understandings from field studies with considerations of the potential roles of robots as a first step of design. By prioritizing situated considerations over techno-solutionism–an approach that sees design as mere problem solving and optimization without regard for potential consequences when implemented in a context that is in fact more complex–our work can contribute to more nuanced and successful design approaches in Human-Robot Interaction. We are optimistic that our presented findings, considerations and approach can serve as starting points for future work aimed at developing novel roles for robotic technologies aligned with values of care.

## Data Availability

The datasets presented in this article are not readily available because of the sensitive nature of our research context and agreements with participants. Requests to access the datasets should be directed to RV, ralf.vetter@plus.ac.at.

## Footnotes

1 Version 2022.0.0, https://www.maxqda.com/products/maxqda
